# Supplementary figures and images for: A brain-wide form of presynaptic active zone plasticity orchestrates resilience to brain aging in Drosophila
Source: PLoS Biol. 2022 Dec 5;20(12):e3001730. doi: 10.1371/journal.pbio.3001730 (PMC9721493; doi:10.1371/journal.pbio.3001730)

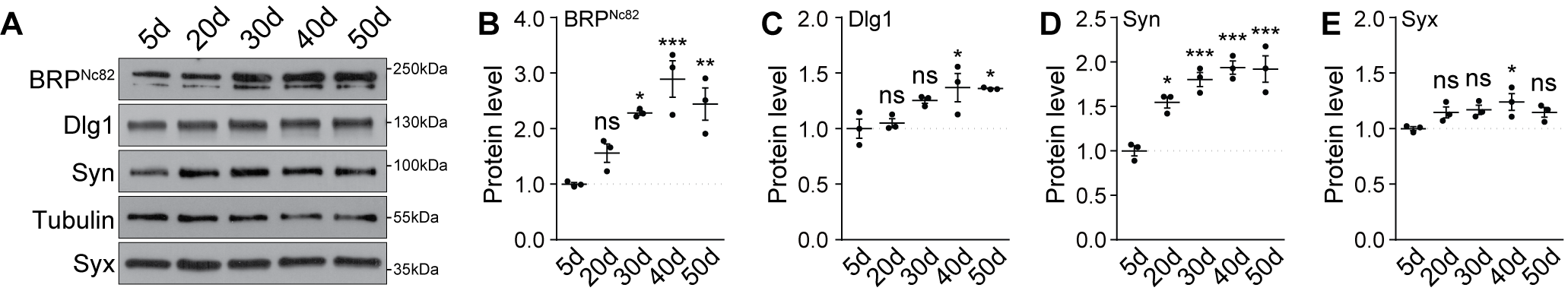

Supplement: S1 Fig — (A-E) Representative western blots (A) and statistics of a spectrum of synaptic proteins, including BRP Nc82 (B), Dlg1 (C), Syn (D), and Syx (E) in wt female flies with aging. n = 3. One-way ANOVA with Bonferroni multiple comparisons test is shown. *p < 0.05; **p < 0.01; ***p < 0.001; ns, not significant. Error bars: mean ± SEM. Underlying data can be found in S1 Data Sheet. Raw images of this figure are provided in S1 Raw Images. Dlg1, Discs large; Syn, Synapsin; Syx, Syntaxin; wt, wild type. (TIF) [file pbio.3001730.s001.tif]

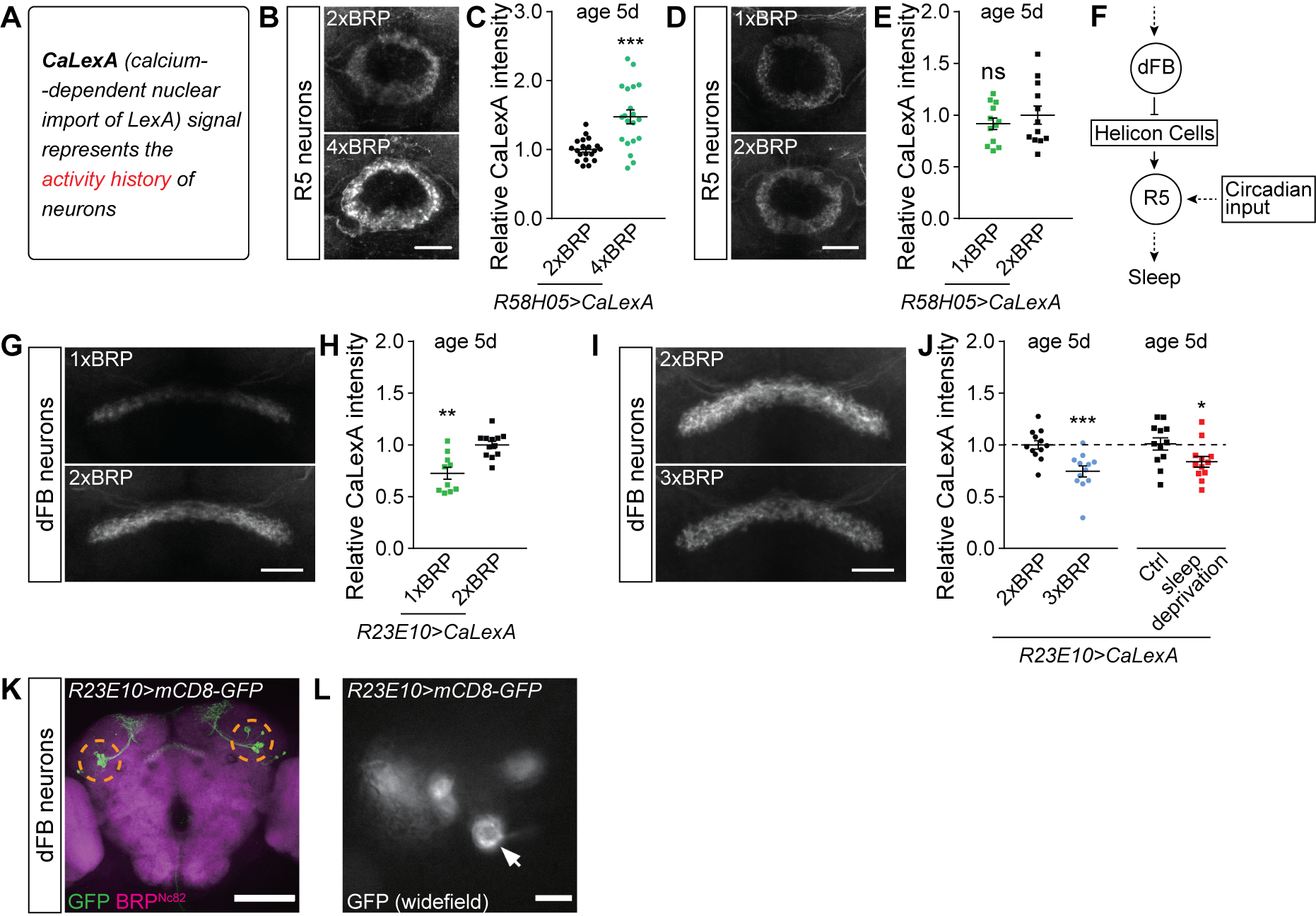

Supplement: S2 Fig — (A) CaLexA signal is likely the representation of activity history of neurons. (B and C) Confocal images (B) and whole-mount brain staining analysis (C) of CaLexA signal intensity with CaLexA expressed in R5 neurons by R58H05-Gal4 in 4xBRP compared to 2xBRP flies. n = 20 for all groups. (D and E) Confocal images (D) and whole-mount brain staining analysis (E) of CaLexA signal intensity with CaLexA expressed in R5 neurons by R58H05-Gal4 in 1xBRP compared to 2xBRP flies. n = 12 for all groups. (F) Scheme of an interconnected sleep circuit in the central complex composed of the dFB (marked by R23E10-Gal4), Helicon cells, and the ellipsoid body R5 neurons (R5 marked by R58H05-Gal4). R5 neurons also receive circadian inputs. (G and H) Confocal images (G) and whole-mount brain staining analysis (H) of CaLexA signal intensity with CaLexA expressed in R5 neurons by R23E10-Gal4 in 1xBRP compared to 2xBRP flies. n = 10–12. (I and J) Confocal images (I) and whole-mount brain staining analysis (J) of CaLexA signal intensity with CaLexA expressed in R5 neurons by R23E10-Gal4 in 1xBRP compared to 2xBRP flies, and in sleep-deprived flies. Sleep deprivation was performed between ZT12 to ZT24 during nighttime. n = 12 for all groups. Student t test is shown. *p < 0.05; **p < 0.01; ***p < 0.001; ns, not significant. Scale bar: 20 μm. Error bars: mean ± SEM. (K) Confocal image of the expression pattern of R23E10-Gal4 indicated by GFP staining. Scale bar: 100 μm. (L) Widefield image of the cell bodies of R23E10-Gal4 indicated by live GFP signal, the cell body localization is indicated in the dashed circles of (J). Scale bar: 10 μm. Underlying data can be found in S1 Data Sheet. dFB, dorsal fan-shaped body; ZT, zeitgeber time. (TIF) [file pbio.3001730.s002.tif]

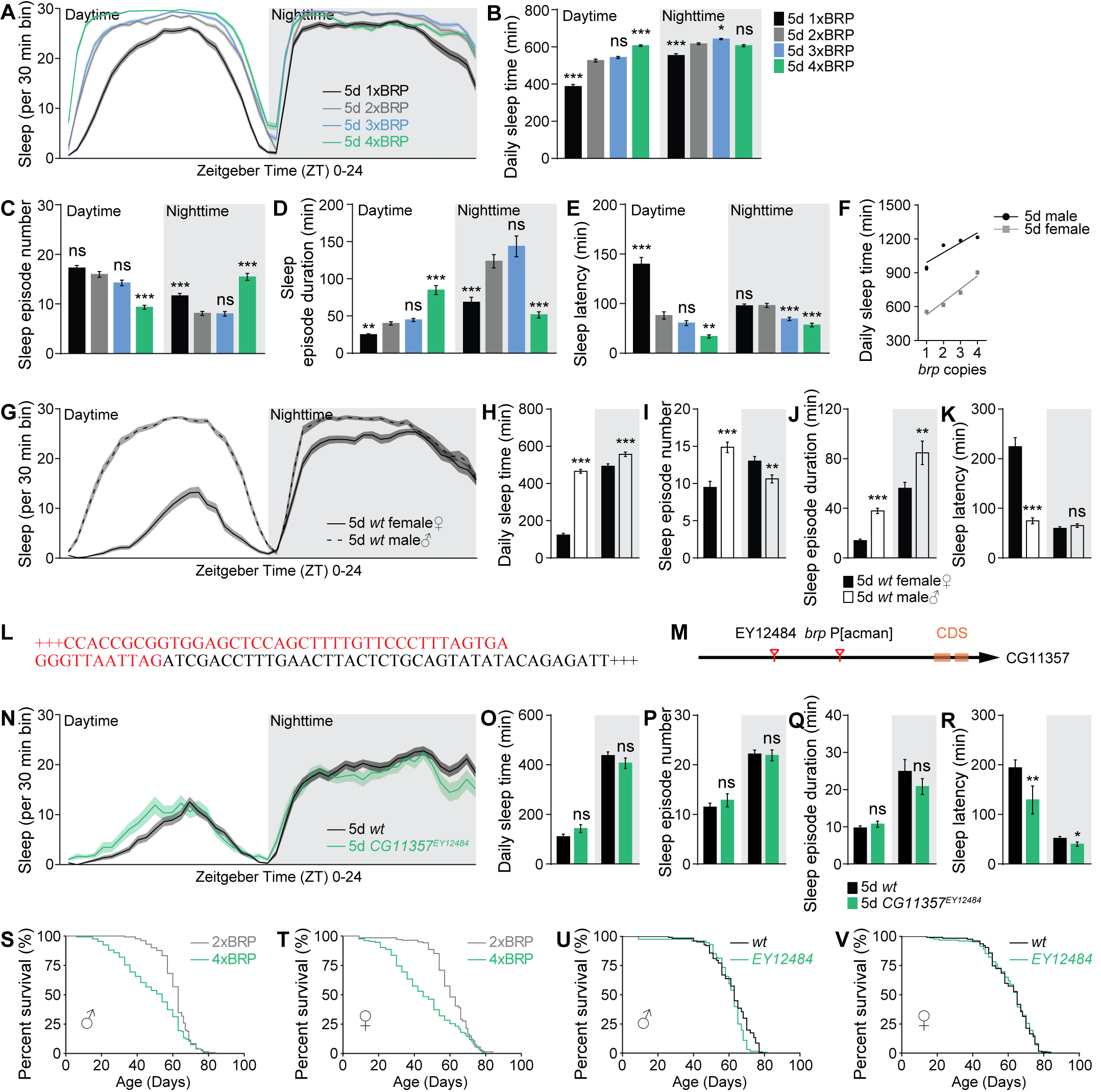

Supplement: S3 Fig — (A-E) Sleep structure of 1xBRP-4xBRP male flies averaged from measurements over 2–4 days, including sleep profile plotted in 30-min bins (A), daily sleep amount (B), number and duration of sleep episodes (C and D), and sleep latencies (E). n = 63–80. One-way ANOVA with Bonferroni multiple comparisons test is shown. (F) Linear regression analysis of daily sleep amount in flies with different brp copies in both male (R2 = 0.81) and female (R2 = 0.95) flies. n = 63–80 per group for male, n = 123–128 per group for female. (G-K) Sleep structure of 5d female and male wt flies from measurements over 2–4 days, including sleep profile plotted in 30-min bins (G), daily sleep amount (H), number and duration of sleep episodes (I and J), and sleep latencies (K). n = 62–64 per group. (L) Genomic mapping and sequence of the integration site of the brp P[acman] transgenic construct. Red letters indicate brp P[acman] sequence, and black letters indicate genomic CG11357 gene sequence. (M) Simplified gene span of CG11357 and the integration site of the brp P[acman] and another P-element mediated allele EY12484 that are both localized at the 5′ UTR region of CG11357. (N-R) Sleep structure of EY12484 female flies averaged from measurements over 2–4 days, including sleep profile plotted in 30-min bins (N), daily sleep amount (O), number and duration of sleep episodes (P and Q), and sleep latencies (R). n = 55 for wt control and n = 32 for EY12484. Student t test is shown. (S and T) An independent experiment of the lifespan analysis of 2xBRP and 4xBRP flies. For male flies (S), n = 134 for 4xBRP compared to 2xBRP (n = 132, p < 0.001). For female flies (T), n = 134 for 4xBRP compared to 2xBRP wt control flies (n = 141, p < 0.001). (U and V) Lifespan analysis of 2xBRP wt and EY12484 flies. For male flies (U), n = 128 for EY12484 compared to 2xBRP (n = 127, ns). For female flies (V), n = 129 for EY12484 compared to 2xBRP wt control flies (n = 127, ns). Gehan–Breslow–Wilcoxon test is shown fo [file pbio.3001730.s003.tif]

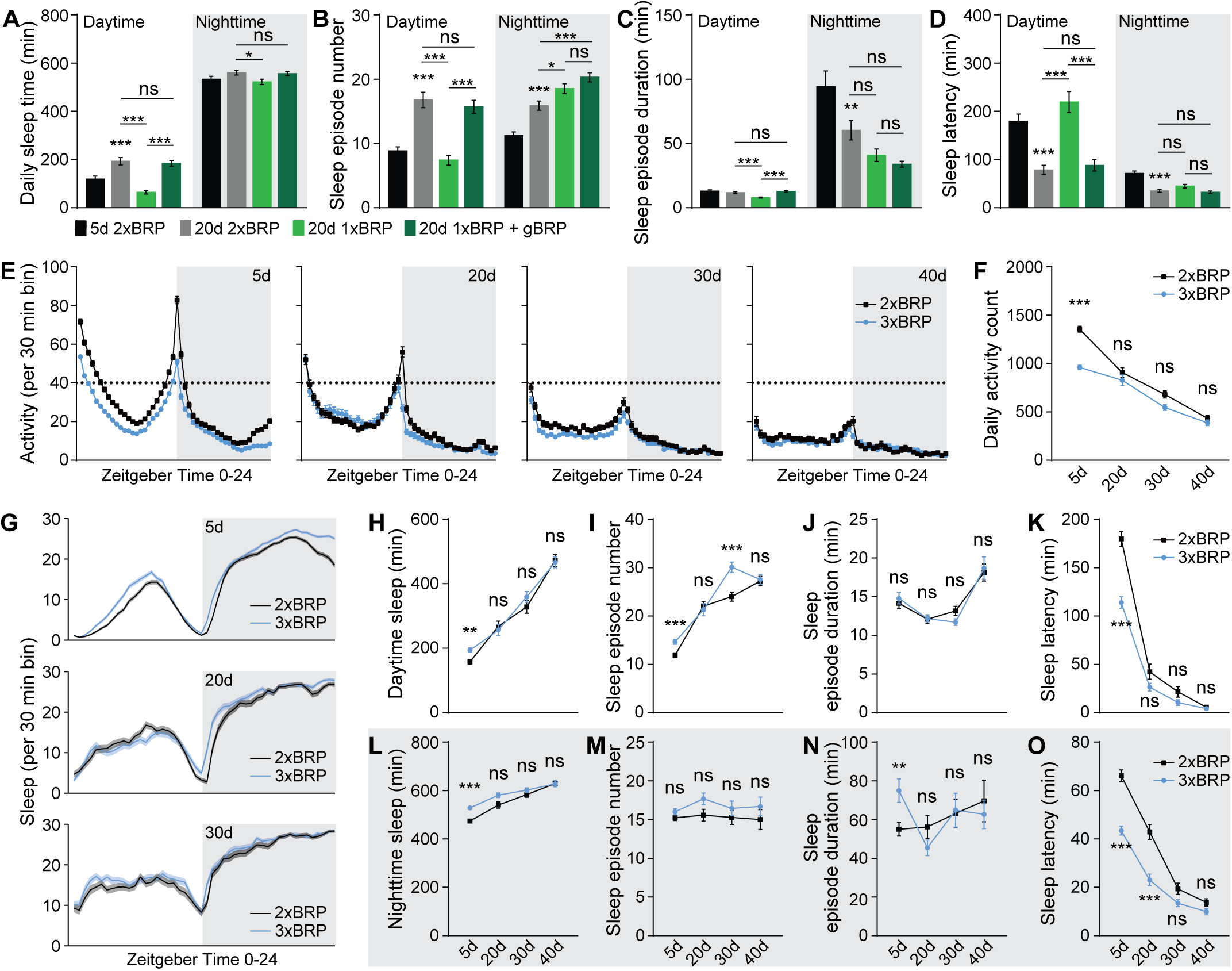

Supplement: S4 Fig — (A-D) Sleep structure of 1xBRP female flies at age 20d rescued by a transgenic brp copy (gBRP) and averaged from measurements over 2–3 days, including daily sleep amount (A), number and duration of sleep episodes (B and C), and sleep latencies (D). n = 63–64 for all groups. One-way ANOVA with Bonferroni multiple comparisons test is shown. (E and F) Locomotor walking activity distribution across the day (E) and averaged daily total walking activity (F) of 3xBRP compared to 2xBRP female flies at ages 5d, 20d, 30d, and 40d. (G-O) Sleep structure of 3xBRP female flies at ages 5d, 20d, 30d, and 40d averaged from measurements over 2–3 days, including sleep profile plotted in 30-min bins (G), daytime and nighttime sleep amount (H and L), number and duration of sleep episodes (I, J, M, and N), and sleep latencies (K and O). n = 246–247 for 5d, n = 61–63 for 20d, n = 59–62 for 30d, and n = 32 for 40d. Two-way ANOVA with Sidak multiple comparisons is shown. *p < 0.05; **p < 0.01; ***p < 0.001; ns, not significant. Error bars: mean ± SEM. Underlying data can be found in S1 Data Sheet. (TIF) [file pbio.3001730.s004.tif]

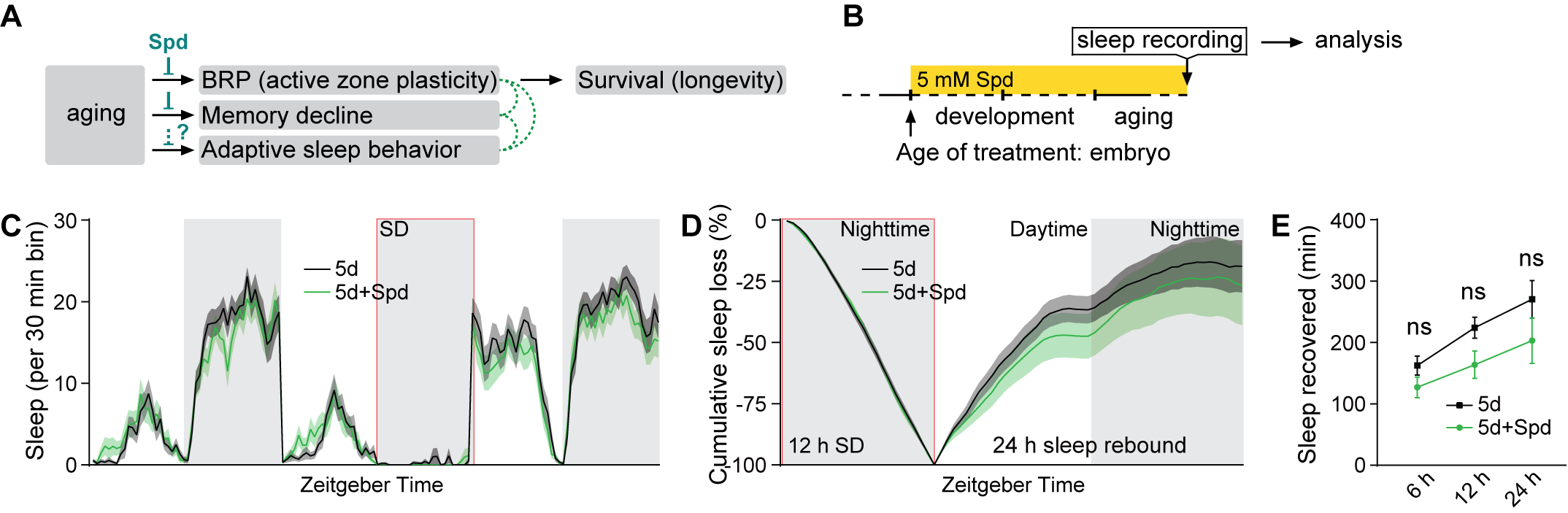

Supplement: S5 Fig — (A and B) Rationale (A) and protocol (B) for the consequence of Spd supplementation in age-associated alterations of sleep pattern. Early aging trigger PreScale, memory decline, and sleep pattern changes, which might functionally intersect for survival. Spd supplementation was shown to suppress PreScale and memory decline, but its effect on early aging-associated sleep pattern changes was unclear. (C) Sleep profile for 5d wt female flies treated with 5 mM Spd compared to untreated for 3 consecutive days. (D) Normalized cumulative sleep loss during 12-h nighttime sleep deprivation and 24-h sleep rebound. Two-way repeated-measures ANOVA with Fisher LSD test did not detect any significant treatment × time interaction (F(47, 2784) = 0.0038; p > 0.9999) during sleep rebound. (E) Sleep recovered at three different time points after sleep deprivation for 5d 5 mM Spd-treated compared to untreated female flies. n = 29–31 for both groups. Two-way ANOVA with Sidak multiple comparisons is shown. ns, not significant. Error bars: mean ± SEM. Underlying data can be found in S1 Data Sheet. LSD, least significant difference; Spd, spermidine; wt, wild type. (TIF) [file pbio.3001730.s005.tif]

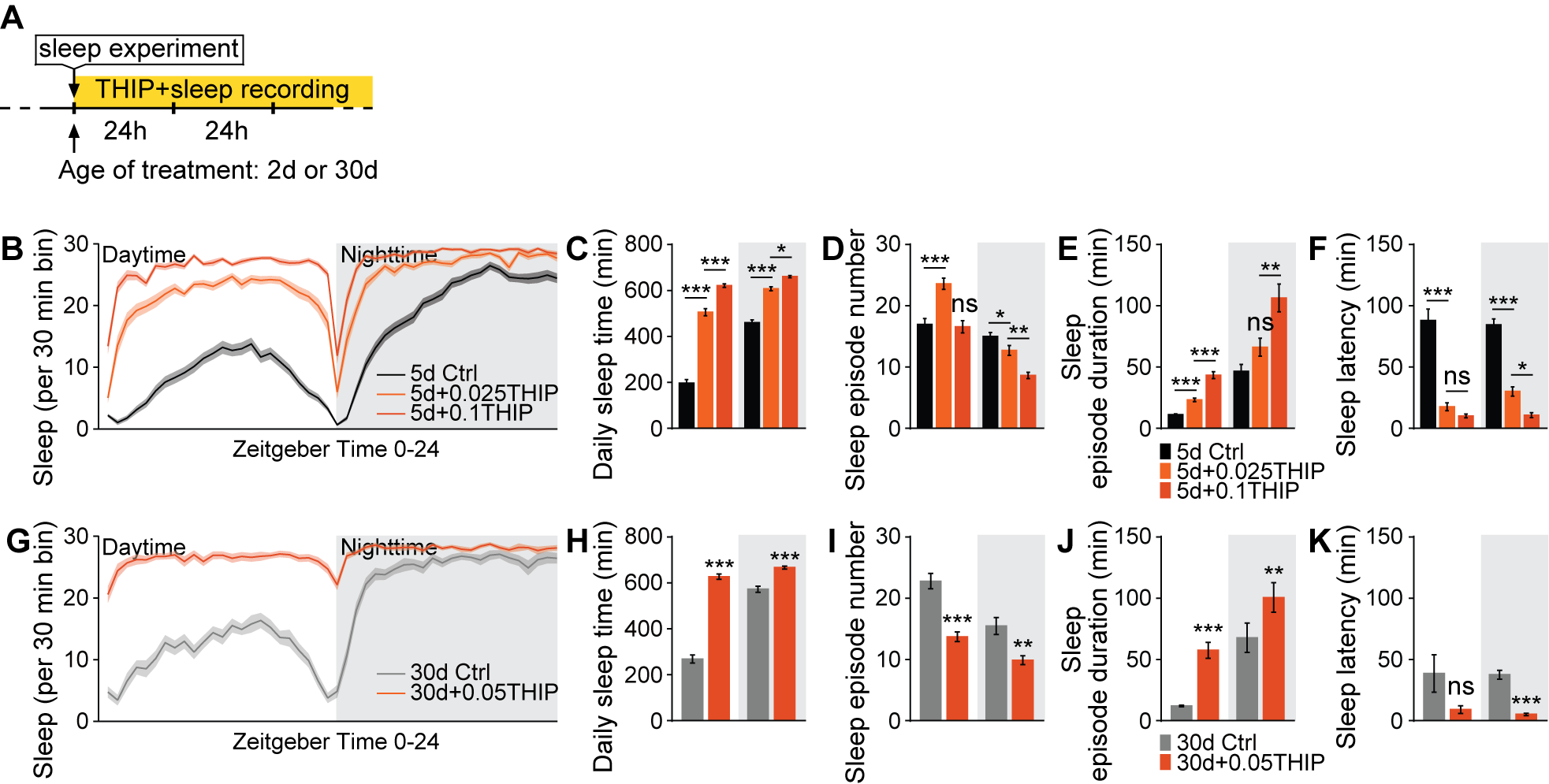

Supplement: S6 Fig — (A) Protocol for sleep test of wt female flies treated with different concentrations of THIP at either age 3d or 30d. (B-F) Sleep structure of 5d wt female flies fed with 0.05 mg ml−1 and 0.1 mg ml−1 THIP from measurements over 2–3 days, including sleep profile plotted in 30-min bins (B), daily sleep amount (C), number and duration of sleep episodes (D and E), and sleep latencies (F). n = 64 for untreated control wt flies, n = 32 for both 0.05 mg ml−1 and 0.1 mg ml−1 THIP-treated groups. One-way ANOVA with Bonferroni multiple comparisons test is shown. (G-K) Sleep structure of 30d wt female flies fed with 0.05 mg ml−1 THIP from measurements over 2–3 days, including sleep profile plotted in 30-min bins (G), daily sleep amount (H), number and duration of sleep episodes (I and J), and sleep latencies (K). n = 31 for both groups. Student t test is shown. *p < 0.05; **p < 0.01; ***p < 0.001; ns, not significant. Error bars: mean ± SEM. Underlying data can be found in S1 Data Sheet. (TIF) [file pbio.3001730.s006.tif]

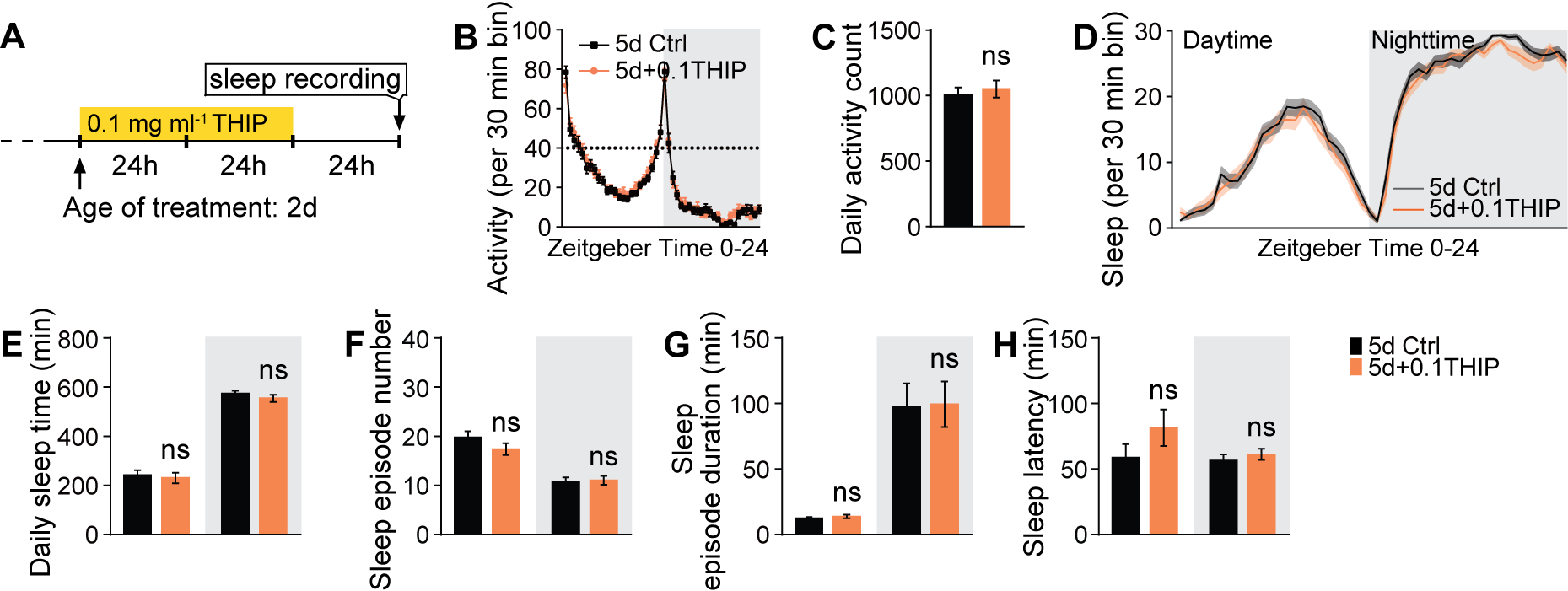

Supplement: S7 Fig — (A) Protocol for sleep test of wt female flies that have been treated with 0.1 mg ml−1 THIP for 2 days at age 2d. (B and C) Locomotor walking activity pattern (B) and statistic (C) of 30d wt female flies after 2 days of 0.1 mg ml−1 THIP treatment. (D-H) Sleep structure of 2d wt female flies after 2 days of 0.1 mg ml−1 THIP treatment averaged from measurements over 2 days, including sleep profile plotted in 30-min bins (D), daytime and nighttime sleep amount (E), number and duration of sleep episodes (F and G), and sleep latencies (H). n = 32 for all groups. Student t test is shown. ns, not significant. Error bars: mean ± SEM. Underlying data can be found in S1 Data Sheet. (TIF) [file pbio.3001730.s007.tif]

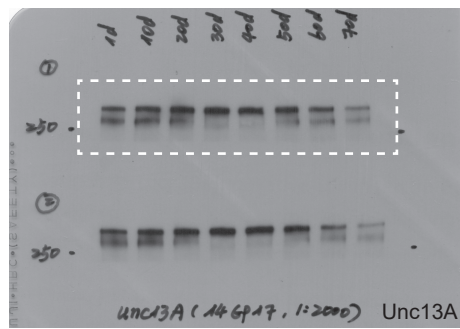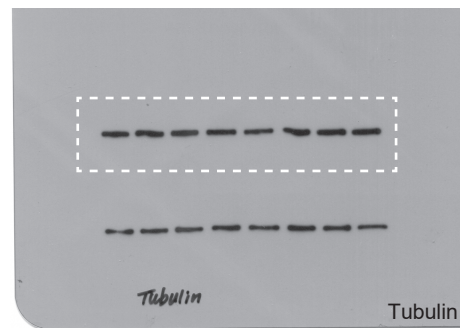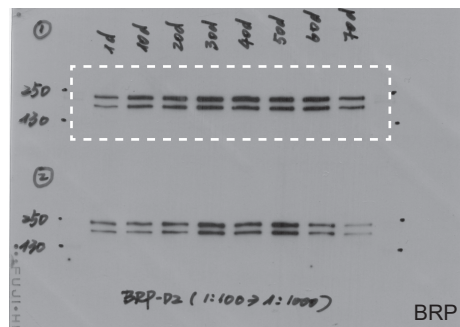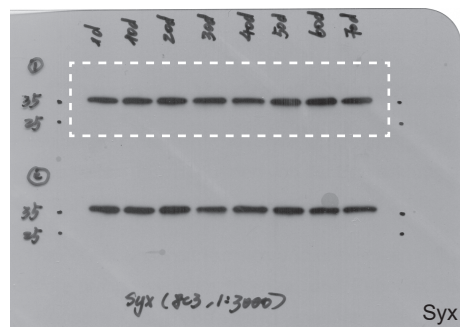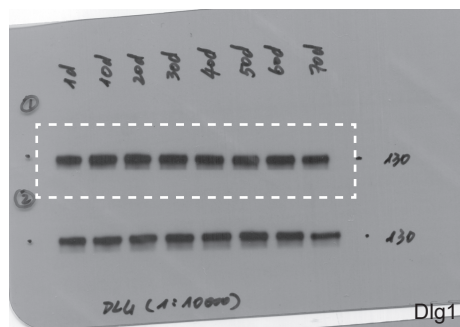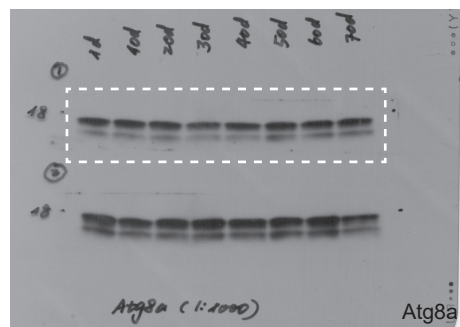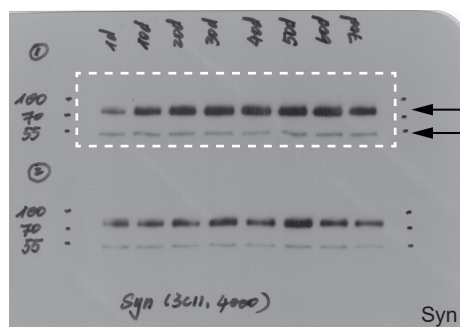

Fig 1B

- ① replicate 1 or gel 1
- ② replicate 2 or gel 2

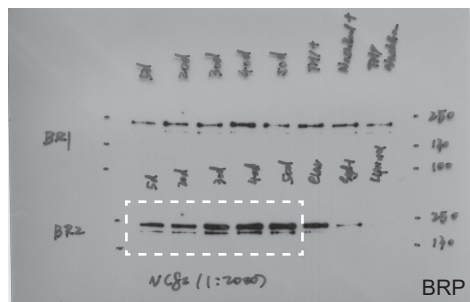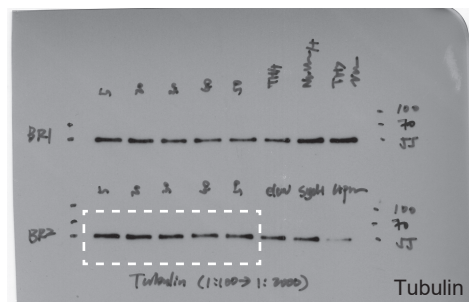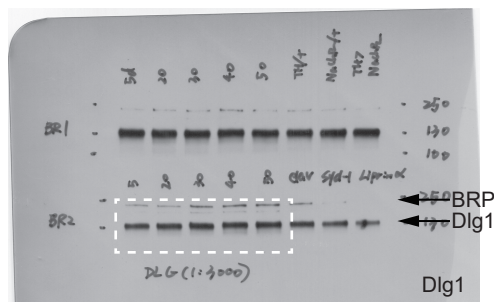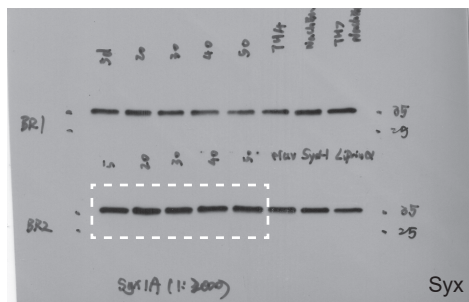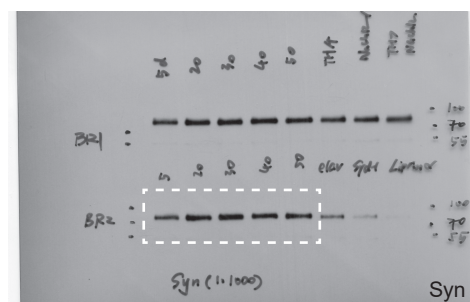

S1A Fig

BR1: replicate 1 or gel 1

BR2: replicate 2 or gel 2

Supplement: S1 Raw Images — (PDF) [file pbio.3001730.s016.pdf]
