# Supplementary material for: A brain-wide form of presynaptic active zone plasticity orchestrates resilience to brain aging in Drosophila
Source: PLoS Biol. 2022 Dec 5;20(12):e3001730. doi: 10.1371/journal.pbio.3001730 (PMC9721493; doi:10.1371/journal.pbio.3001730)
Supplement: Attachment — Submitted filename: Huang et al Plos biology R1 rebuttal final.pdf [file pbio.3001730.s017.pdf]

Responses to Reviewers

We appreciate the very valuable comments of the reviewers on our manuscript. However, the reviewers also raise important issues on how to improve our manuscript. In the following, we address their specific remarks.

REVIEWS:

Reviewer #1: Huang et al. characterize a presynaptic phenomenon called PreScale and propose that age-dependent induction of PreScale may be responsible for regulating lifespan and age-related changes in sleep and memory. Overall, their manuscript is somewhat disorganized, the presentation of their data seems haphazard, and the description of their model is difficult to understand. Unfortunately, the amount of progress in this work compared to previously published work from this group is unclear.

First of all, we would like to express our deep gratitude to the reviewer for having carefully read and commented on our manuscript in an insightful manner. We believe that this allowed for substantially improving our revised manuscript. In response to the following comments, we performed a series of new experiments and elaborated our interpretations and model.

The hypothesis of behavioral adaptations executed by PreScale during early aging we propose here is based on both biochemical and behavioral experiments. While studies mostly focus on advanced age, how the early phase of aging affects behavior is often neglected, which is taken into consideration in this study. We propose that early aging-associated behavioral alterations might well be adaptive and survival-protective. Indeed, while our manuscript was under revision at *PLOS Biology*, a study published at *Nature Aging* show that rapamycin treatment in early adulthood suffices to extend lifespan, but not during advanced aging [1].

We previously proposed the causal role of PreScale in memory decline during early aging [2], and the causal role of PreScale in encoding sleep need after sleep loss [3], however, our understanding towards the mechanistic and conceptual actions of PreScale was very limited. We here further examined the role of PreScale in greater details and depth. Based on these findings, we now propose a new, fundamentally different hypothesis identifying PreScale as a novel molecular plasticity program that steers trade-offs and adaptions during early aging (Reviewer figure 1 and manuscript Figure 8).

We propose that PreScale is formed ”on demand” in response to a neuronal physiological state switch which builds in response to aging as well as to sleep deprivation. PreScale then coordinates to favor longevity over new memory formation. Our data suggest that THIP treatment and spermidine supplementation are able to reset an age-induced physiological state responsible for triggering PreScale. In effect, PreScale and THIP treatment make PreScale unnecessary and dispensable (Reviewer figure 1 and manuscript Figure 8).

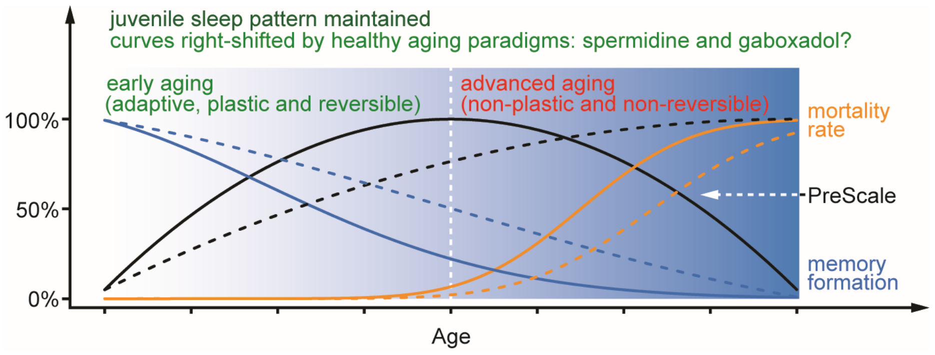

Reviewer figure 1. A model for PreScale in executing trade-offs between sleep homeostasis, memory formation and longevity (from manuscript Figure 8).

The solid curves represent normal aging, the right-shifted dashed curves are the aging process under spermidine (Spd) supplementation or Gaboxadol (THIP) treatment.

We provide the following *new data* in our revised manuscript:

1) We measured the sleep pattern of flies after THIP treatment (manuscript Figures 7E-7L);

2) We performed epistatic experiments showing that PreScale is causal for memory improvement in mid-aged flies (manuscripts Figure 7M-7P);

3) We performed memory experiments showing that flies with advanced age (50-day-old) were no longer improved by THIP treatment, compared to flies during early aging (30-day-old) (manuscript Figures 6Q-6R). These data support the model that early aging phase is likely adaptive, plastic and reversible, while advanced aging phase might be non-plastic and non-reversible.

4) We examined the effects of 1xBRP on dFB and R5 utilizing CaLexA system (manuscript Figure S2).

Please allow us to highlight the major advances of our current work (also including the data incorporated during the revision) over our previous findings:

i) We for the first time provide a detailed analysis of presynaptic active zone plasticity (PreScale) across the whole lifespan of *Drosophila* (manuscript Figures 1 and S1).

ii) We provide evidence that PreScale-type synaptic plasticity suffices to provoke early aging-associated sleep pattern changes (manuscript Figures 4, 5 and 7).

iii) We show that spermidine supplementation, previously shown to extend lifespan, promote memory formation and suppress PreScale in mid-aged animals, does also attenuate the early-age-associated sleep pattern changes (manuscript Figures 5 and S5) which we report in the current study.

iv) To decipher a role of age-associated sleep pattern changes alongside early aging, we used an acute treatment with sleep-promoting Gaboxadol (THIP). We show that THIP treatment has similar consequences as we have observed for spermidine supplementation: extended lifespan, improved memory formation and suppressed PreScale (manuscript Figures 6 and 7). We further show that the suppressed PreScale is causal for memory improvement after THIP treatment in mid-aged flies (manuscript Figure 7). Thus, both paradigms seemingly converge into a similar scenario allowing for a reset along the early aging trajectory.

v) We performed *in vivo* patch-clamp electrophysiology of the sleep- and memory-promoting dorsal fan-shaped body (manuscript Figure 2) and provide the first cellular mechanistic explanations for PreScale in triggering early aging-associated sleep pattern changes, memory decline and longevity.

Concerning the organization of our manuscript, we started by explicitly characterizing synaptic plasticity across the fly lifespan (manuscript Figure 1), followed by cellular and mechanistic examination at the dFB neurons for the comparison between genetic PreScale mimicry (4xBRP) and early aging-associated PreScale (in 20-day-old animals, manuscript Figure 2). Afterwards, we provided detailed analysis of the survival and behavioral-relevance of PreScale during early aging (manuscript Figures 3 and 4). We then analyzed two rejuvenation paradigms (spermidine/Spd supplementation and Gaboxadol/THIP treatment) in coupling with PreScale-type plasticity and early aging-associated memory decline (manuscript Figures 5-7). We hope that our manuscript is now more comprehensive and the idea that PreScale plays a central role in coupling sleep homeostasis, memory and longevity is delivered more clearly.

Specific comments:
1) The Introduction is short and vague. The first two paragraphs, which make up the bulk of the Introduction, are general background, and the important third paragraph, which introduces PreScale and spermidine, is very brief, making it difficult to understand what PreScale is and why it is important. Because the Introduction is short, much introductory material is inserted into the Results section making the logic of the manuscript more difficult to follow. Without first reading Gupta et al., 2016 and Huang et al., 2020, many readers may have difficulty understanding the rationale behind most of the experiments in the current manuscript. In fact, the authors reference a supplementary figure in Huang et al., 2020, requiring readers to look up this figure. A better introduction to these papers may improve the organization and apparent significance of this manuscript.

We deeply appreciate this comment. In the revised manuscript, we thoroughly introduced our previous work in Introduction. Please refer to the lines 55-67.

2) The model presented by the authors is vague, and the interactions between sleep, lifespan, memory and PreScale are unclear. For example, in the graphic abstract and in Figure S7, both spermidine and gaboxadol decrease PreScale, improve memory and extend lifespan, but they have opposite effects on sleep. Do the

authors think that PreScale and sleep are unrelated? This seems unlikely since this group published Huang et al., 2020. The partial models presented in Figures S5A and 7A are difficult to understand. They seem to suggest that if PreScale is inhibited, sleep is reduced. On the other hand, if sleep is increased, PreScale is inhibited. These seem to be opposing effects that the authors could discuss more clearly.

We apologize for having being unclear and somewhat confusing at this point.

For spermidine, we examined the consequences of its treatment in early aging-associated sleep pattern changes, meanwhile memory is retained and PreScale is inhibited.

For Gaboxadol/THIP, it is strongly sleep-promoting *during* acute treatment (manuscript Figure S6). Effects on the sleep behavior of *Drosophila* *after* having applied THIP were not reported so far. We also examined the consequences of THIP treatment (i.e. *after* THIP treatment). We now in the revised version show that indeed 30d *wt* flies exhibited less sleep when tested *after* THIP treatment, similar to the effects of spermidine supplementation (Reviewer figure 2, now also incorporated into manuscript Figure 7). This effect is specific to 30d aged flies, as 5d flies did not show difference in sleep *after* THIP treatment (manuscript Figure S7).

We now tried to graphically bundle our findings and interpretations in the proposed model (GRAPHIC ABSTRACT and manuscript Figure 8).

We now arrive at a very similar relation concerning the effects of acute Gaboxadol/THIP and spermidine/Spd supplementation in mid-aged 30d animals: suppressed PreScale, protected ability to form new memories, as well as increased lifespan. We speculate that THIP treatment and spermidine supplementation might be able to reset the neuronal physiological state during early aging and thus make PreScale dispensable (Reviewer figure 1).

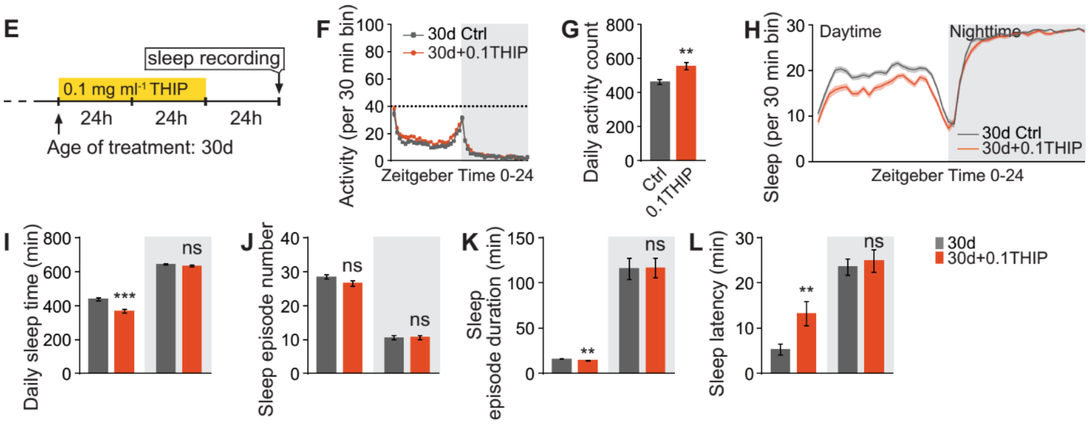

Reviewer figure 2. Sleep pattern changes were suppressed after THIP treatment, similar to spermidine treatment (from manuscript Figure 7).

30-day-old (30d) *wt* flies were treated with 0.1 mg ml^{-1} THIP (0.1THIP) for 2 days. The locomotor activity and sleep were performed 1 day after the treatment.
(**E**) Protocol for sleep test of *wt* female flies which have been treated with 0.1 mg ml^{-1} THIP for two days at age 30d.
(**F** and **G**) Locomotor walking activity pattern (**F**) and statistic (**G**) of 30d wt flies after 2 days of 0.1 mg ml-1 THIP treatment.
(**H-L**) Sleep structure of 30d wt female flies after 2 days of 0.1 mg ml-1 THIP treatment averaged from measurements over 2 days, including sleep profile plotted in 30-min bins (H), daytime and nighttime sleep amount (**I**), number and duration of sleep episodes (**J** and **K**), and sleep latencies (**L**). n = 90-94 for all groups. Student's t test is shown** $p < 0.01$; *** $p < 0.001$; ns, not significant. Error bars: mean ± SEM.

Concerning the comment "*They seem to suggest that if PreScale is inhibited, sleep is reduced. On the other hand, if sleep is increased, PreScale is inhibited*": this apparent paradox between sleep and PreScale has been addressed in our recent publication [3]: sleep reduces PreScale, while sleep loss promotes PreScale to encode sleep need in order to ultimately reduce PreScale itself (Reviewer figure 3).

Again we should have described our model in a more concise and also graphical form. A simple model of the relations we found is shown below (Reviewer figure 3). As said, we extended the Introduction in order to succinctly describe our previous findings in the revised manuscript now (Please refer to the lines 55-67).

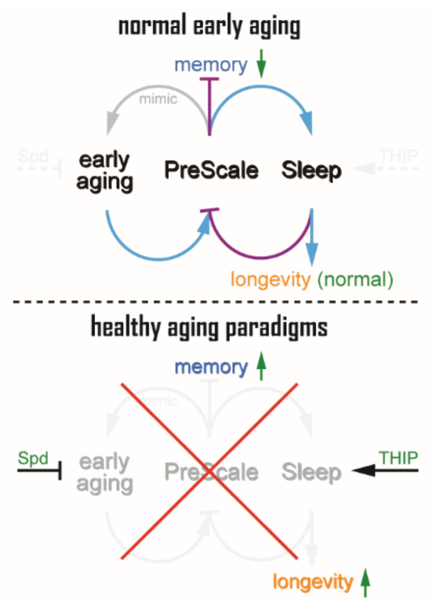

Reviewer figure 3. A simplified model depicting the roles of PreScale in executing the trade-offs between memory formation and longevity (from manuscript Figure 8).

The emergence of PreScale during normal early aging executes the trade-offs between memory and longevity: suppressed memory probably for normal longevity (upper panel). Interventions like spermidine (Spd) and Gaboxadol (THIP) during early aging likely eliminate the need for such trade-offs, and promote both memory and longevity (lower panel).

3) Many of the results in this manuscript are correlative, and causative relationships are unclear. For example, both spermidine and gaboxadol inhibit PreScale. However, they have opposing effects on sleep, suggesting that at least for one of these interventions, effects on sleep are not mediated by PreScale. Since sleep is known to be linked to memory and lifespan, it is unclear whether the effects of e.g. gaboxadol on lifespan and memory are caused by changes in PreScale or by changes in sleep. Epistatic experiments addressing causation were not performed.

The reviewer is definitely right that establishing causal relations in the aging context is challenging, also given the interconnected nature of the phenotypic readouts. In this manuscript, we exploited and further established a genetic mimicry of early aging-associated PreScale (by solely tuning *brp* gene copy number), which we found to allow for phenocopying several aspects of sleep deprivation- and early aging-provoked changes in young flies. Still, as correctly expressed by the reviewer, a challenge but also chance here is to establish epistatic relations between this form of plasticity and the relevant behavioral components.

To explicitly test an epistatic relation in the revision of our manuscript, we examined the effects of genetically triggering PreScale (by increasing BRP copies from 2xBRP to 3xBRP) when interfering with the effects of THIP treatment on memory at 30d. We reasoned that, if the suppression of PreScale (measured via brain BRP levels) was indeed causally connected with the post-THIP-observed memory improvement in 30d flies, increasing BRP copies from 2xBRP to 3xBRP might attenuate the post-THIP memory effects. This is indeed what we found: THIP did improve memory in 2xBRP but not 3xBRP flies at the age of 30d (Reviewer figure 4). Notably, THIP did not increase memory at 5d in neither 2xBRP nor 3xBRP (Reviewer figure 4). These data are now also incorporated into manuscript Figure 7.

These new results thus suggest a causal connection between suppressing PreScale and restoring memory formation in mid-aged flies.

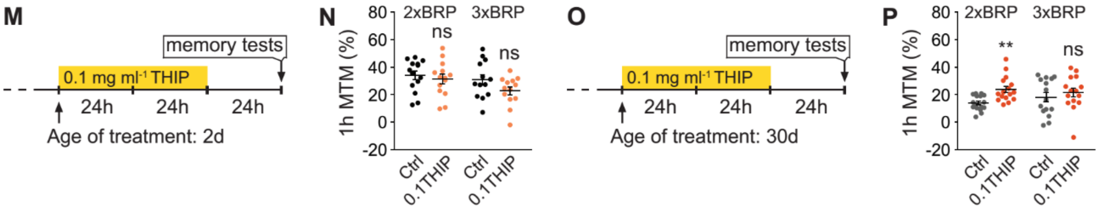

Reviewer figure 4. Increasing BRP copies from 2xBRP to 3xBRP blocks the effect of THIP on memory during early aging (from manuscript Figure 7).

2-day-old (2d) or 30d 2xBRP and 3xBRP flies were treated with 0.1 mg ml^{-1} THIP (0.1THIP) for 2 days (**M** and **O**). 1h MTM experiment was performed 1 day after the treatment (**N** and **P**). n = 15-16 for all groups. One-way ANOVA with Bonferroni's multiple comparisons test is shown. ** $p < 0.01$; ns, not significant. Error bars: mean ± SEM.

4) Did the authors examine the effects of age-dependent induction of *bruchpilot* RNAi on sleep, lifespan, and memory? Does this increase age-dependent lifespan and memory and maintain young sleep amounts?

This is surely a valuable suggestion. Obviously, age-specific *brp* RNAi induction experiments need a post-developmental, temporally well-defined knockdown scenario without interfering a normal aging process. Thus, we decided to not use the Gal80^{ts} system, which depends on temperature shifts from 18°C to 29°C, a procedure which change the aging process, sleep behavior and many other aspects. Instead, we in the context of this revision performed a first experiment using the GeneSwitch system [4]. In our pilot experiments towards this question, we used *elav-GeneSwitch(GS)-Gal4* to induce *brp* RNAi at the age of 30d by feeding the flies with the inducer RU486. As shown below, feeding 30d flies with 1mM RU486 for 5 days did provoke a slight trend in reducing sleep (Reviewer figure 5). Obviously, a systematic analysis here would need an extensive characterization of the kinetics of BRP downregulation in response to the induction.

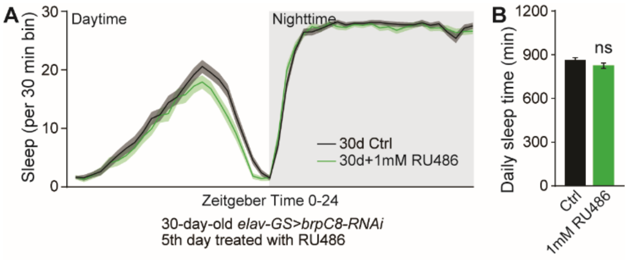

Reviewer figure 5. *brp-RNAi* during adult stage at age 30d does not obviously alter sleep behavior.

elav-GS>brpC8-RNAi flies were aged on normal food to the age 30d. The 30d female flies were then loaded onto either 1mM RU486 or control sleep food for sleep recording. The sleep of the 5th day was analyzed and shown. n = 64 per group. Student's t test is shown. ns, not significant. Error bars: mean ± SEM.

5) Did the authors examine the effects of 1xBRP on dFB and R5? Are these effects the opposite of 3xBRP?

This is a very interesting question. To answer this, we examined CaLexA signals in dFB and R5 of 1xBRP flies at the age of 5d. While increasing BRP copies from 2x to 3x and 4xBRP upregulates R5 neuronal activity, decreasing BRP copies from 2xBRP to 1xBRP did not change the activity of these neurons (Reviewer figure 6, now also incorporated into manuscript Figure S2).

Interestingly, we found that both increasing (3xBRP and 4xBRP) and decreasing (1xBRP) BRP copies reduced the neuronal activity of the dFB (Reviewer figure 6, now also incorporated into manuscript Figure S2). We have recently shown that genetically installing PreScale by increasing *brp* gene copy number mimics sleep-deprived animals, while reducing brp gene copy number provokes a slight reduction in sleep [3]. We would argue that the lower CaLexA signal of dFB represents the mild sleep reduction in 1xBRP flies [3]. For 3xBRP to 4xBRP flies, reduced CaLexA signal might represent a mimicry of sleep-deprived flies (manuscript Figures S2I and S2J). As CaLexA signal is a proxy concerning the activity history of neurons, the lower activity of dFB thus likely reflects the sleep history of sleep deprivation [5]. Indeed, we also found that sleep-deprived flies showed lower CaLexA signal (Reviewer figure 6, now also incorporated into manuscript Figure S2). We also discussed these results in the revised manuscript (Please refer to the lines 143-152 and 466-477).

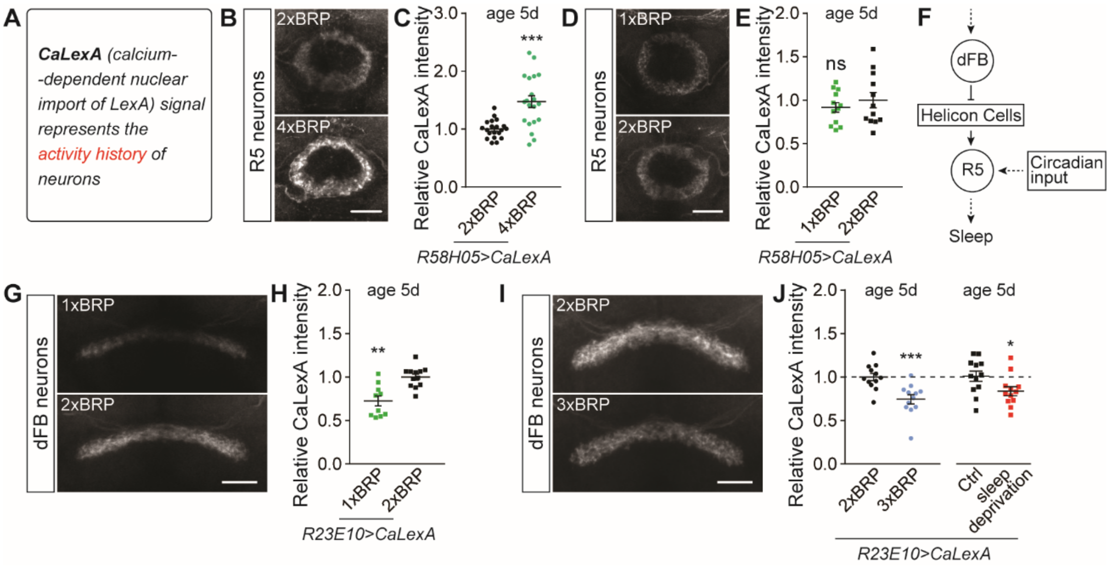

Reviewer figure 6. PreScale regulates the neuronal activity of sleep-regulating R5 and dFB neurons (from manuscript Figure S2).

(**A**) CaLexA signal is likely the representation of activity history of neurons [5].
(**B** and **C**) Confocal images (**B**) and whole-mount brain staining analysis (**C**) of CaLexA signal intensity with CaLexA expressed in R5 neurons by R58H05-Gal4 in 4xBRP compared to 2xBRP flies. n = 20 for all groups.
(**D** and **E**) Confocal images (**D**) and whole-mount brain staining analysis (**E**) of CaLexA signal intensity with CaLexA expressed in R5 neurons by R58H05-Gal4 in 1xBRP compared to 2xBRP flies. n = 12 for all groups.
(**F**) Scheme of an interconnected sleep circuit in the central complex composed of the dorsal fan-shaped body (dFB, marked by *R23E10-Gal4*), Helicon cells and R5 neurons (R5 marked by *R58H05-Gal4*). R5 neurons also receive circadian inputs.
(**G** and **H**) Confocal images (**G**) and whole-mount brain staining analysis (**H**) of CaLexA signal intensity with CaLexA expressed in R5 neurons by R23E10-Gal4 in 1xBRP compared to 2xBRP flies. n = 10-12.
(**I** and **J**) Confocal images (**I**) and whole-mount brain staining analysis (**J**) of CaLexA signal intensity with CaLexA expressed in R5 neurons by R23E10-Gal4 in 1xBRP compared to 2xBRP flies, and in sleep-deprived flies. n = 12 for all groups. Student's t test is shown. *$p < 0.05$; **$p < 0.01$; ***$p < 0.001$; ns, not significant. Scale bar: 20 µm. Error bars: mean ± SEM.

6) How spontaneous activity and membrane excitability of dFB neurons are affected by spermidine and gaboxadol?

We thank the reviewer for suggesting these experiments. Unfortunately, patch clamp *in vivo* electrophysiological recordings in the adult *Drosophila* brain are really challenging, especially for dFB neurons whose cell bodies are particularly small. As also found in rodents, increasing age makes patch clamp recordings even more challenging. As we described in the first submission, it was almost impossible to record 30-day-old dFB neurons and, despite efforts, we only could record from a few 20-day-old animals in the original submission (Please refer to the lines 168-171). It is unfortunately impossible to finish these experiments in a time frame reasonable for the revision of our manuscript.

As Reviewer #2 suggested to analyze the dFB electrophysiology of young 5d 3xBRP flies, we decided to start these experiments and managed to patch a few 5d 3xBRP dFB neurons. These neurons were all in their OFF state [6], which means they hardly fired action potentials in response to current injections (Reviewer figures 7A-7B). Still, similar to 4xBRP animals (manuscript Figure 2J), 5d 3xBRP animals showed a clear reduction in input resistance (Reviewer figure 7C), indicating a reduced excitability in dFB neurons also under 3xBRP. The finding that more dFB neurons in OFF state was not specific to 3xBRP, as in our previous experiments many of the dFB neurons in 5d 4xBRP animals were also in OFF state, indicative of a *per se* reduced excitability of the dFB neurons in flies with increasing BRP copy number. These observations suggest that 3xBRP and 4xBRP are essentially similar in dFB electrophysical properties (Reviewer figure 7 and manuscript Figure 2J), consistent with the CaLexA results (manuscript Figures 2C and 2D).

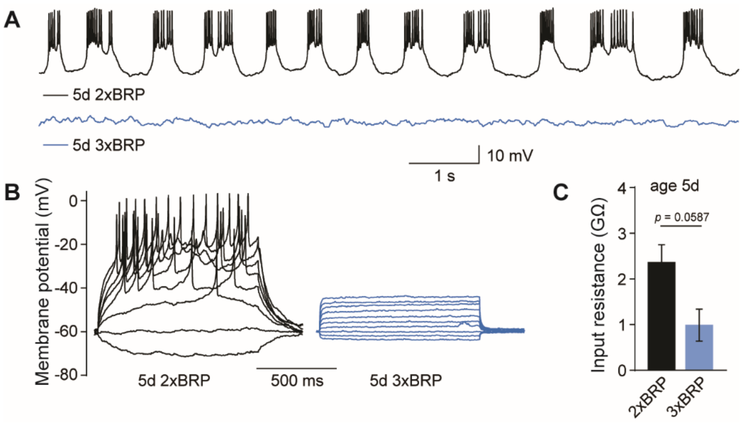

Reviewer figure 7. Example of the electrophysiological properties of 5d 3xBRP dFB neurons in OFF state.

OFF state dFB neurons lack of spontaneous spikes (**A**), and current injections did not elicit action potentials (**B**). Though we were not able to analyze most of the electrophysiological parameters in OFF state dFB neurons, we observed a clear decrease in the input resistance of 5d 3xBRP dFB neurons (**C**), similar to 4xBRP animals (manuscript Figure 2J). n = 3 per group. Student's t test is shown. Error bars: mean ± SEM.

In our manuscript, we provided cellular evidence that genetically induced PreScale and early aging-associated PreScale do result in a qualitatively similar scenario (manuscript Figure 2). We wanted to provide cellular evidence of genetically-encoded PreScale in mimicking early aging process, in addition to our behavioral analysis. Due to the great amount of work and time needed to finish these experiments, and the focus of our manuscript being on the role of PreScale in steering trade-offs during early aging, a detailed, extensive physiological consequence of both spermidine and Gaboxadol effects is unfortunately beyond the scope of our current study.

Reviewer #2: Huang et al. examined age-associated changes in synaptic protein levels and neuronal excitability, as well as sleep, memory, and longevity of flies with genetically manipulated BRP levels or those treated with spermidine or THIP. The results are potentially interesting, but I have several major concerns regarding the conceptual framework, data interpretation, and experimental design. A primary concern is that they do not explore sex differences in their data and do not examine reproductive fitness as a critical factor in a fly's life. Moreover, the authors conclude that "a brain-wide form of presynaptic active zone plasticity ("PreScale") promotes resilience by coupling sleep, longevity and memory during aging (abstract)." However, while the manuscript provides correlative data, it is unclear whether they point to a causal role of PreScale in the coordinated age-associated changes. Specific concerns are listed below.

We thank this reviewer for in depth reviewing our work and finding it *per se* interesting. We deeply appreciate the great suggestions concerning sex differences and reproductive fitness, as we also wanted to thoroughly examine the roles of PreScale in various aspects of organismal aging.

We admit that establishing causal relations in the aging context is challenging, also given the interconnected nature of the phenotypic readouts. As mentioned above and in the revised manuscript, the genetic mimicry of early aging-associated PreScale by tuning *brp* gene copy number is a paradigm we developed that phenocopies many aspects of sleep-deprived flies and the flies during early aging [2, 3], and in our opinion renders itself to establish causal and epistatic relationships also between the behavioral entities along the aging trajectory.

Along those lines, and also reflecting a similar comment from reviewer #1, we now in the revision time examined the effects of genetically triggered PreScale (by *brp* gene copy number to from 2xBRP to 3xBRP) when interfering with the effects of THIP treatment on memory at 30d. We reasoned that, if the suppression of PreScale indicated by BRP down-regulation was indeed causal for the memory improvement in 30d flies, increasing *brp* gene copy number from 2xBRP to 3xBRP might attenuate the effect of THIP in memory. This was indeed the case: THIP did improve memory in 2xBRP but not 3xBRP flies at the age of 30d (Reviewer figure 4, now also incorporated into manuscript Figure 7). Notably, THIP did not increase memory at 5d in

neither 2xBRP nor 3xBRP (Reviewer figure 4, now also incorporated into manuscript Figure 7). These new results indeed suggest a causal connection of suppressing PreScale in memory restoration in mid-aged flies.

1. In what sense is the tradeoff between longevity and memory during early aging optimal? What is optimized? It seems that simply living longer wouldn't be the core purpose of a fly. One could argue that producing lots of healthy progeny would be the fly's core mission. The authors should examine reproductive fitness (e.g., # of offspring) as a critical aspect of the necessary tradeoffs as flies age.

We sincerely thank the reviewer for this great suggestion. We now tested the fecundity of 1-4xBRP female flies along aging. We found that 1xBRP, 2xBRP and 3xBRP flies were not different at most ages, except 50d at which 1xBRP flies were obviously lower in the eggs produced per female (Reviewer figure 8). 4xBRP flies, however, were always lower in the amount of eggs produced per female at each age tested (As shown in manuscript Figure 3A, BRP level in 5d 4xBRP was already higher than reached in the context of normal aging).

However, we consider the 3xBRP scenario as a physiological mimicry of the extent of normal early aging-associated PreScale (manuscript Figures 1 and 3A). As the fecundity of 3xBRP was not reduced versus 2xBRP (but at the same time 3xBRP lives longer), 3xBRP animals might indeed be able to produce more offspring in a lifetime (however, under challenging, variable conditions, 2xBRP with its more effective memory might be advantageous). Thus, a moderate extent of PreScale during early aging (mimicked by 3xBRP) seems obviously to not undermine fecundity (Reviewer figure 8). For the moment being, and given the somewhat limited extent of our fecundity analysis, we would prefer to not include the data in the revised manuscript. However, if the reviewer has different opinion on this decision, we would be prepared to include these data.

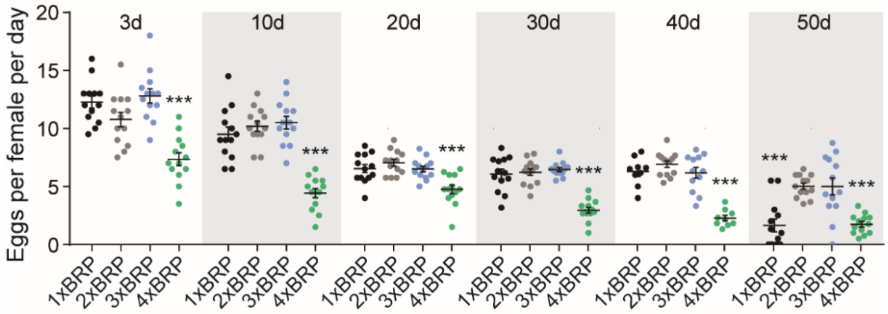

Reviewer figure 8. Fecundity of 1x-4xBRP female flies along aging.

Female flies were sorted into small vials with normal food 2 days in advance of their age indicated in Reviewer figure 8. Depending on the ages, the number of female flies within each vial was different. 3 female for age 3d, ~4 for 10d and 20d, ~6 for 30/40/50d. In each vial of the whole experiment, two 5d *wt* male flies were included. Sorted flies were transferred into fresh normal food at zeitgeber time (ZT) 3, and were allowed to lay eggs for 24 hours. Afterwards, the amount of eggs in each vial was manually counted. n = ~13 per group. One-way ANOVA with Bonferroni's multiple comparisons test is shown. ***$p < 0.001$. Error bars: mean ± SEM.

Here, under our normal stable laboratory rearing condition of aging process within which the lifespan is often maximized, we propose that a balance between longevity and memory formation is optimized by PreScale, likely through the regulation of sleep pattern changes during early aging. Living longer in stable laboratory rearing conditions, while accompanying with compromised costly behaviors (e.g. memory formation) without undermining reproductive success, could certainly allow for more egg production and finally more progenies in a fly's life. In this sense, the trade-offs between memory and longevity executed by PreScale during early aging without reducing reproductive fitness might actually be optimal.

2. The data show substantial sex differences (e.g., Fig. 3), but there is no serious discussion of the subject. As mentioned in Point #1, the authors should examine how reproductive success is impacted by various genetic and drug manipulations.

We appreciate this comment. We discussed the sex difference in the revised manuscript. Please refer to the lines 199-204 and 363-366.

3. The authors propose that PreScale promotes resilience during early aging by favoring longevity over memory. The finding that 3xBRP promotes longevity at the expense of memory seems consistent with the view. However, Spd and THIP promote both memory and longevity while suppressing PreScale. If PreScale has adaptive advantages, why do spermidine and THIP, which suppress PreScale, improve both memory and

longevity? The authors suggest that Spd and THIP treatment promote efficient mitochondrial electron transport and autophagy, making PreScale unnecessary. How can we tell a priori whether a particular PreScale manipulation will enhance both memory and longevity or favor one over the other? What is meant by "a specific, context-dependent role of PreScale in regulating lifespan (Line 182)." Please elaborate on the specific contexts. If wake and inc mutants already have increased BRP, why does it help to have even more?

We appreciate discussing these considerations and clarifying our perspective. We propose that Spd and THIP treatment *eliminate the need* for the occurrence of PreScale during early aging, leading to the absence of trade-offs between memory and longevity, e.g. both memory and longevity are promoted. From the extended longevity and suppressed memory of 3xBRP animals (and the stable, unchanged fecundity of 3xBRP versus 2xBRP), it seems that PreScale primarily favors survival (and subsequently a likely somewhat higher reproductive success) over "costly" memory [7, 8]. Manipulations reducing PreScale (Spd supplementation and THIP treatment) thus allowed to "reopen the window" for the formation of new memories (Reviewer figure 1 and manuscript Figure 8). It is certainly very interesting to understand how exactly Spd and THIP extend lifespan without PreScale. We speculate that Spd and THIP might steer metabolic reprograming to regulate lifespan.

In Huang et al., 2020, we proposed that increasing BRP upon sleep loss in *wake* and *inc* mutants is a protective action to counteract sleep loss (for example by promoting sleep). Introducing an additional *brp* copy thus help the mutants in this direction.

The statement "*a specific, context-dependent role of PreScale in regulating lifespan*" was based on the results that 3xBRP promotes longevity in *wt*, *wake* and *inc* mutant backgrounds, but not in *atg7* mutant and in an AD model context (it could well be that autophagy is downstream of PreScale). In this sense, it might be explained by a role of autophagy for PreScale in promoting lifespan.

4. The increased longevity due to PreScale and THIP treatment could be because they sleep more and therefore spend less energy. The disadvantage may be that they do not have the opportunity to produce many offspring. Again, if the authors consider reproductive fitness, the increased longevity accompanied by increased sleep may not be a good tradeoff.

We thank the reviewer for triggering this discussion. As we also discussed in the original submission (lines 427-434), we fully agree with the reviewer that sleep-promoting PreScale and THIP treatment save energy during sleep for promoting lifespan. However, 3xBRP animals did not show any difference in fecundity during aging (Reviewer figure 8). Thus, a likely physiological mimicry of the extent of normal early aging-associated PreScale in 3xBRP animals with extended lifespan seems advantageous in terms of overall reproductive fitness, at least under the conditions chosen.

5. Fig. 2 shows striking physiological effects of increasing BRP levels to 4x. However, sleep and longevity data suggest that whereas 3xBRP provides some beneficial effects similar to PreScale, 4xBRP is detrimental. Given this finding, they should examine the electrophysiological properties for 3xBRP, not 4xBRP.

We thank the reviewer for suggesting these experiments. Even though patch clamp *in vivo* electrophysiological recordings in the adult *Drosophila* brain are really challenging, especially for dFB neurons whose cell bodies are particularly small, we managed to patch a few 5d 3xBRP dFB neurons. These neurons were all in their OFF state [6], which means they hardly fired action potentials in response to current injections (Reviewer figure 9). Still, similar to 4xBRP animals (manuscript Figure 2J), 5d 3xBRP animals showed a clear reduction in input resistance (Reviewer figure 9C), indicating a reduced excitability in dFB neurons also under 3xBRP. The finding that more dFB neurons in OFF state was not specific to 3xBRP, as in our previous experiments many of the dFB neurons in 5d 4xBRP animals were also in OFF state, indicative of a *per se* reduced excitability of the dFB neurons in flies with increasing BRP copy number. These observations suggest that 3xBRP and 4xBRP are essentially similar in dFB electrophysical properties (Reviewer figure 9 and manuscript Figure 2J), consistent with the CaLexA results (manuscript Figures 2C and 2D).

Taken together, as both 3xBRP and 4xBRP flies show increased sleep at age 5d [3] and they all show significantly reduced CaLexA signal in dFB neurons (manuscript Figures 2C and 2D), we believe that 3xBRP flies are likely similar to 4xBRP in dFB electrophysiology at young age 5d.

Furthermore, we tend to believe that, at young age 5d when the mortality rate is as low as 2xBRP *wt*, 4xBRP could mimic mid-aged animals in sleep pattern and memory impairment [2]. Only during aging process, the BRP level might be too high in 4xBRP, exceeding the maximum of normally aging 2xBRP *wt* animals. Thus, the similar *in vivo* dFB electrophysiological properties in 5d young 4xBRP and 20d aged 2xBRP animals support the notion of PreScale mimicking the naturally occurring PreScale during early aging by either 3xBRP or 4xBRP.

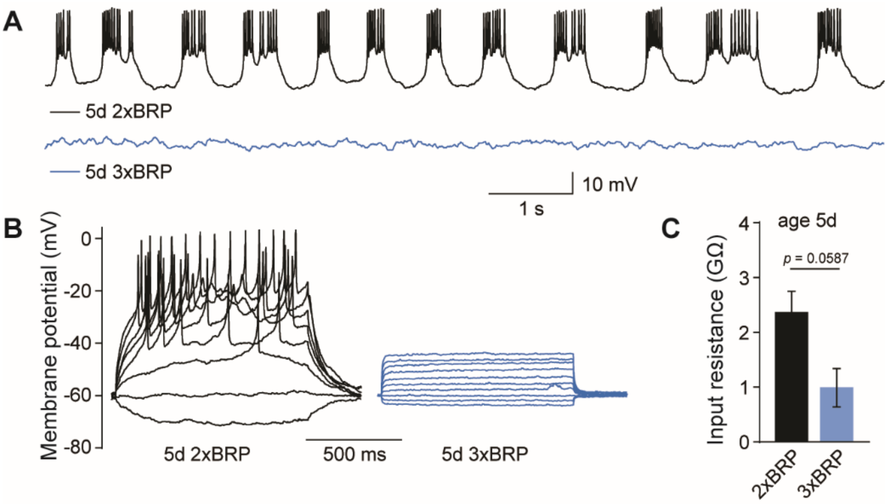

Reviewer figure 9. Example of 5d 3xBRP dFB neurons in OFF state which was lack of spontaneous spikes (A), and current injections did not elicit action potentials (B).

OFF state dFB neurons lack of spontaneous spikes (**A**), and current injections did not elicit action potentials (**B**). Though we were not able to analyze most of the electrophysiological parameters in OFF state dFB neurons, we observed a clear decrease in the input resistance of 5d 3xBRP dFB neurons (**C**), similar to 4xBRP animals (manuscript Figure 2J). n = 3 per group. Student's t test is shown. Error bars: mean ± SEM.

6. Why does PreScale have opposite effects on the neuronal excitability of R5 vs. dFB?

R5 and dFB are negatively connected via helicon cells, forming a relaxation circuit for loading and unloading/ dispensing sleep need [9].

We would like to emphasize that, the circuit formed by dFB-Helicon-R5 neurons is not isolated in the brain circuit complex. For example, dFB neurons receive inputs from wake-promoting dopaminergic neurons [6]. In addition, a circadian output circuit innervates R5 neurons to regulate sleep [10, 11]. Thus, the activity of dFB and R5 neurons might represent a complex physiological state which integrates different input information to coordinate animals behaviors.

In addition, sleep deprivation, which triggers PreScale [3, 12], increases the activity of R5 neurons [13] but dampens the activity of dFB neurons (Reviewer figure 6J and manuscript Figure S2J) measured via CaLexA. Thus, genetically triggered PreScale (3xBRP and 4xBRP) again mimicked sleep-deprived animals.

We also discussed these results in the revised manuscript (Please refer to the lines 478-490).

7. In the model (graphical abstract and Fig. S7), they show "longevity" as something affected by aging. Since longevity stays constant across the lifespan, mortality, which increases with aging, would be more helpful.

Very good suggestions! We followed these thoughts in our revised manuscript. As for the model, we present it in a more comprehensive and simplified manner in the revised manuscript (Graphical abstract and manuscript Figure 8).

8. The effects of THIP on memory seem variable across experiments. For example, 0.1THIP had no effect on STM in one experiment (H) but a significant effect in another (N). The effects of 3xBRP on longevity are also variable across experiments (Fig. 3C vs. E). What accounts for the differences?

We are happy to clarify this point. STM in manuscript Figure 6H was tested *immediately after* 0.1THIP treatment, while STM in manuscript Figure 6N was tested *one day after* 0.1THIP treatment. This is indeed an observation we emphasized already in the original version (lines 319-323 and 326-327). Please also refer to the difference in protocol in manuscript Figure 6E and Figure 6M.

Why such a single day gap led to memory improvement we indeed consider as very interesting and should followed up in further studies.

9. Why do synaptic protein levels decline after middle age? Do older flies remember better after PreScale returns to the young fly level?

This is indeed a very interesting consideration, as *per se* the decline in PreScale might indeed favor learning and memory.

We propose in the manuscript that early aging until middle age represents a still plastic phase and PreScale adds on adaptivity here allowing to favor better survival over memory formation (Reviewer figure 1 and manuscript Figure 8). However, after middle age with increased mortality rates, flies might enter into a non-plastic phase (Reviewer figure 1 and manuscript Figure 8). We suggest that in advanced age, additional factors might become rate-limiting, e.g. metabolic reprogramming, mitochondrial function, and autophagy status.

Indeed, to potentially answer this questions, we performed memory experiments on 50d *wt* flies after 2-day THIP treatment, compared to 30d animals. We show that such a treatment can no longer reverse memory defects in 50d flies (Reviewer figure 10). It will be very interesting to identify these additional factors and processes limiting the reversibility of memory formation in advanced age in future studies.

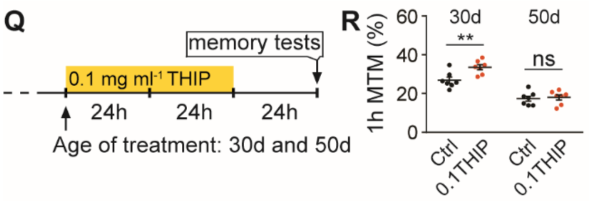

Reviewer figure 10. THIP treatment benefits 30d *wt* flies in 1h MTM, but not 50d *wt* flies (from manuscript Figure 6).

30-day-old (30d) and 50d *wt* flies were treated with 0.1 mg ml^{-1} THIP (0.1THIP) for 2 days. 1h MTM experiment was performed 1 day after the treatment compared to untreated siblings. n = 7 per group. One-way ANOVA with Bonferroni's multiple comparisons test is shown. $^{**}p < 0.01$; ns, not significant. Error bars: mean ± SEM.

10. Some figure legends say "flies." Please specify whether they are male or female flies.

We appreciate this comment. We used both female and male flies for longevity and memory experiments. Female flies were used for most of the sleep experiments, as they show stronger age-associated sleep pattern changes (lines 684-685 and 748-749). We now at all instances specify the sex of flies in the revised manuscript for experiments with either male for female flies. For memory experiments, we used general ”flies” or ”animals”.

References

1. Juricic P, Lu Y-X, Leech T, Drews LF, Paulitz J, Lu J, et al. Long-lasting geroprotection from brief rapamycin treatment in early adulthood by persistently increased intestinal autophagy. Nature Aging. 2022;2(9):824-36. doi: 10.1038/s43587-022-00278-w.

2. Gupta VK, Pech U, Bhukel A, Fulterer A, Ender A, Mauermann SF, et al. Spermidine Suppresses Age-Associated Memory Impairment by Preventing Adverse Increase of Presynaptic Active Zone Size and Release. PLoS Biol. 2016;14(9):e1002563. doi: 10.1371/journal.pbio.1002563. PubMed PMID: 27684064.

3. Huang S, Piao C, Beuschel CB, Gotz T, Sigrist SJ. Presynaptic Active Zone Plasticity Encodes Sleep Need in Drosophila. Current biology : CB. 2020. Epub 2020/03/07. doi: 10.1016/j.cub.2020.01.019. PubMed PMID: 32142702.

4. Nicholson L, Singh GK, Osterwalder T, Roman GW, Davis RL, Keshishian H. Spatial and temporal control of gene expression in Drosophila using the inducible GeneSwitch GAL4 system. I. Screen for larval nervous system drivers. Genetics. 2008;178(1):215-34. Epub 2008/01/19. doi: 10.1534/genetics.107.081968. PubMed PMID: 18202369; PubMed Central PMCID: PMCPMC2206072.

5. Masuyama K, Zhang Y, Rao Y, Wang JW. Mapping neural circuits with activity-dependent nuclear import of a transcription factor. J Neurogenet. 2012;26(1):89-102. Epub 2012/01/13. doi: 10.3109/01677063.2011.642910. PubMed PMID: 22236090; PubMed Central PMCID: PMCPMC3357894.

6. Pimentel D, Donlea JM, Talbot CB, Song SM, Thurston AJ, Miesenbock G. Operation of a homeostatic sleep switch. Nature. 2016;536(7616):333-7. doi: 10.1038/nature19055. PubMed PMID: 27487216; PubMed Central PMCID: PMCPMC4998959.

7. Placais PY, Preat T. To favor survival under food shortage, the brain disables costly memory. Science. 2013;339(6118):440-2. Epub 2013/01/26. doi: 10.1126/science.1226018. PubMed PMID: 23349289.

8. Placais PY, de Tredern E, Scheunemann L, Trannoy S, Goguel V, Han KA, et al. Upregulated energy metabolism in the Drosophila mushroom body is the trigger for long-term memory. Nature communications. 2017;8:15510. Epub 2017/06/06. doi: 10.1038/ncomms15510. PubMed PMID: 28580949; PubMed Central PMCID: PMCPMC5465319.

9. Donlea JM, Pimentel D, Talbot CB, Kempf A, Omoto JJ, Hartenstein V, et al. Recurrent Circuitry for Balancing Sleep Need and Sleep. Neuron. 2018;97(2):378-89 e4. Epub 2018/01/09. doi: 10.1016/j.neuron.2017.12.016. PubMed PMID: 29307711; PubMed Central PMCID: PMCPMC5779612.

10. Lamaze A, Kratschmer P, Chen KF, Lowe S, Jepson JEC. A Wake-Promoting Circadian Output Circuit in Drosophila. Current biology : CB. 2018;28(19):3098-105 e3. Epub 2018/10/03. doi: 10.1016/j.cub.2018.07.024. PubMed PMID: 30270186.

11. Guo F, Holla M, Diaz MM, Rosbash M. A Circadian Output Circuit Controls Sleep-Wake Arousal in Drosophila. Neuron. 2018;100(3):624-35 e4. Epub 2018/10/03. doi: 10.1016/j.neuron.2018.09.002. PubMed PMID: 30269992.

12. Gilestro GF, Tononi G, Cirelli C. Widespread changes in synaptic markers as a function of sleep and wakefulness in Drosophila. Science. 2009;324(5923):109-12. doi: 10.1126/science.1166673. PubMed PMID: 19342593; PubMed Central PMCID: PMCPMC2715914.

13. Liu S, Liu Q, Tabuchi M, Wu MN. Sleep Drive Is Encoded by Neural Plastic Changes in a Dedicated Circuit. Cell. 2016;165(6):1347-60. doi: 10.1016/j.cell.2016.04.013. PubMed PMID: 27212237; PubMed Central PMCID: PMCPMC4892967.
